# The therapeutic potential of medicinal mushrooms in ophthalmology: from ethnomycology to evidence-based applications

**DOI:** 10.3389/fphar.2026.1887133

**Published:** 2026-09-14

**Authors:** Tibor Rák, Yu Jin, Meirong Chen, Adrienne Csutak

**Affiliations:** 1 Department of Ophthalmology, University of Pécs Medical School, Clinical Centre, Pécs, Hungary; 2 Ophthalmology Department of Shandong Hospital of Traditional Chinese Medicine, Jinan, Shandong Province, China

**Keywords:** antioxidant, immunomodulatory, medicinal mushrooms, ophthalmology, Traditional Chinese medicine

## Abstract

Medicinal mushrooms have long been used in traditional medical systems, yet their ophthalmic relevance has only recently been explored. This narrative review provides, to our knowledge, an integrative mapping of medicinal mushroom-derived bioactive compounds in ophthalmology, with particular emphasis on their level of evidence across different ocular diseases. A structured search of PubMed, Scopus, Google Scholar and ScienceDirect (2001–2026) was conducted to identify experimental, clinical, and mechanistic studies addressing antioxidant, anti-inflammatory, immunomodulatory, neuroprotective and antifibrotic pathways in ocular tissues. The findings reveal a marked heterogeneity in evidence levels. A limited subset of fungal-derived compounds - primarily antimicrobial and immunomodulatory agents such as β-lactam antibiotics, cyclosporine A, and mycophenolate mofetil - are supported by high-level clinical evidence (Level 1), while emerging evidence (Level 2) is available for compounds such as *Isaria cicadae* in dry eye disease and *Ganoderma lucidum* in age-related macular degeneration. In contrast, the majority of medicinal mushrooms - including *Pleurotus* spp., *Hericium erinaceus*, *Wolfiporia extensa*, and *Polyporus umbellatus* - remain supported mainly by preclinical studies and traditional use (Level 5 evidence). Experimental data indicate that mushroom-derived polysaccharides and triterpenoids can modulate oxidative stress, inflammation, intraocular pressure, fibrosis, and neuronal survival. Additional compounds demonstrate potential in glaucoma, cataract, and retinal disorders through multitarget mechanisms. Overall, medicinal mushrooms represent a promising but still under-validated therapeutic resource in ophthalmology. The observed disparity between traditional use, experimental evidence, and clinical validation highlights a significant translational gap, underscoring the need for standardized formulations and well-designed clinical trials to enable evidence-based integration into ophthalmic practice.

## 1 Introduction

Fungi are a unique kingdom of eukaryotic organisms that include yeasts, molds, and macroscopic fruiting body–forming species commonly referred to as mushrooms (Wijayawardene et al., 2024). Medicinal mushrooms have been an integral part of traditional medicine across various cultures for centuries and continue to play a role in modern pharmacology (Rák and Csutak, 2024a). The medicinal mushroom market is experiencing significant growth: in 2024, the market was valued at approximately USD 5.81 billion and is projected to grow to USD 13.67 billion by 2034, with a compound annual growth rate of 8.9% (Singh, 2021). This growth is driven by increasing health consciousness, the rise of veganism, and the expanding use of mushrooms in pharmaceuticals and nutraceuticals.

Historically, fungi have occupied a dual role in ophthalmology, being recognized both as causative agents of severe ocular infections and as therapeutic resources in traditional healing systems (Al-Ghadeer and Al-Amry, 2021; Bautista-González et al., 2022; Knutsson et al., 2023; Rák et al., 2025). The long-standing association of fungi with sight-threatening conditions such as fungal keratitis and endophthalmitis has contributed to their cautious perception in eye care, particularly in regions where ocular mycoses remain a significant cause of visual impairment (Rák et al., 2025). At the same time, empirical ophthalmic applications of medicinal mushrooms are documented in multiple traditional medical systems, including the use of specific species for eye inflammation, visual disturbances, and infectious eye diseases (El Enshasy et al., 2013; Khalifa et al., 2019; Yasin et al., 2019; Panayotov, 2020; N’Douba et al., 2022).

Ancient and medieval medical traditions also described fungus-derived substances influencing vision and visual perception, reflecting early recognition that fungal metabolites can act on neural and retinal pathways (Berlant, 2005; Tipado et al., 2024). Several of these historically used species were later found to contain bioactive compounds with antioxidant, anti-inflammatory, immunomodulatory, and tissue-protective properties, providing a plausible mechanistic basis for their traditional ophthalmic indications (Gouzi et al., 2011; Dahham et al., 2018; Rák and Csutak, 2024a).

With the advent of modern science, the exploration of the therapeutic potential of medicinal mushrooms has expanded, including their application in ophthalmology, particularly in relation to oxidative stress, inflammation, fibrosis, and neurodegeneration as shared pathogenic mechanisms across major eye diseases (Li et al., 2022; Kovács-Valasek et al., 2023; Guo et al., 2024; Rák and Csutak, 2024a). In recent years, increasing attention has been directed toward natural bioactive compounds capable of modulating interconnected molecular cascades involved in ocular pathology.

Despite the growing body of literature on medicinal mushrooms, a clear gap persists in their ophthalmic application. Most previous reviews have focused either on general pharmacological properties or on non-ocular indications, while the ophthalmological relevance of mushroom-derived compounds remains fragmented across experimental, clinical, and ethnomedical sources. Moreover, no prior review has systematically integrated molecular mechanisms (e.g., oxidative stress, inflammation, fibrosis, and neurodegeneration) with specific ocular diseases, while simultaneously considering Traditional Chinese Medicine (TCM) perspectives. This review aims to bridge this gap by providing a comprehensive, mechanism-oriented synthesis of medicinal mushroom research in ophthalmology, integrating evidence from modern biomedical studies and traditional medical systems. The novelty of this review lies in providing an integrative overview specifically focused on ophthalmology, combining molecular, experimental, clinical, and ethnomedical evidence, including TCM-based therapeutic concepts. Furthermore, this work proposes a translational framework linking fungal bioactive compounds to key pathogenic mechanisms in ocular diseases.

## 2 Methodology of data collection

A comprehensive literature search was conducted to identify experimental, clinical and mechanistic evidence on the therapeutic potential of medicinal mushrooms in ophthalmology. The four major databases (PubMed, Scopus, Google Scholar, and ScienceDirect) were systematically screened for publications issued between 1 January 2001, and 31 December 2026. The search strategy combined MeSH terms with free-text keywords, including both scientific and common names of major medicinal mushrooms (e.g., *Ganoderma lucidum*, *Lentinula edodes*, *Cordyceps sinensis*) together with ophthalmic and mechanistic terms such as “ophthalmology,” “ocular diseases,” “dry eye disease,” “cornea,” “glaucoma,” “optic nerve trauma,” “cataract,” “macular degeneration,” “medicinal mushrooms,” “TCM,” “antioxidant,” “immunomodulatory,” “anti-inflammatory,” and “neuroprotective.” Keywords were applied individually and in Boolean combinations to balance sensitivity and specificity.

Eligible studies included original experimental (*in vitro*/*in vivo*), clinical, and observational studies, as well as mechanistic review articles directly focusing on ocular tissues or diseases. Full-text articles published in English and Chinese, and providing sufficient methodological detail were included. To address potential database bias, Chinese-language sources (e.g., CNKI and Wanfang) were also screened where accessible, particularly for studies related to Traditional Chinese Medicine. Study selection was performed independently by two reviewers based on titles, abstracts, and full texts, with disagreements resolved by consensus or senior review. Due to substantial heterogeneity across studies - including differences in mushroom species, bioactive compounds, extraction methods, experimental models, dosing regimens, and outcome measures - a formal systematic review or meta-analysis was not feasible. Therefore, a narrative synthesis approach was adopted to enable integration of diverse evidence types, including experimental findings, clinical observations, and ethnomedical data.

In the following sections, the authors have compiled descriptions of medicinal mushrooms used in evidence-based and Traditional Chinese Medicine ophthalmology to demonstrate their diverse applicability in ophthalmic care. Supplementary Table S1 summarizes the pharmacological properties of medicinal mushrooms with potential ophthalmic relevance. Supplementary Table S1 should therefore be interpreted not merely as a descriptive catalogue, but as a conceptual framework linking specific classes of fungal bioactive compounds to key pathogenic mechanisms in ophthalmology. This framework is further expanded in the following sections, where the compounds are critically evaluated in the context of specific ocular diseases, including their mechanistic relevance and translational potential.

To facilitate the interpretation of these relationships, a schematic overview is provided in Figure 1, reflecting the multitarget nature of these compounds.

**FIGURE 1:**
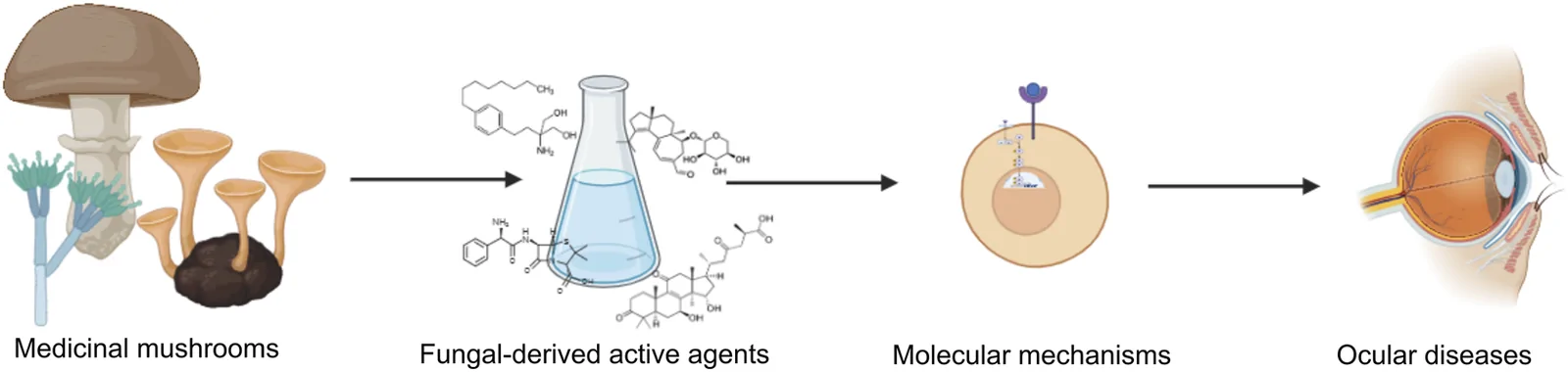
Simplified mechanism-oriented overview of medicinal mushroom-derived bioactive compounds in ophthalmology, linking major compound classes to key pathogenic pathways and ocular diseases. Created with BioRender.com.

## 3 Current mycotherapy opportunities in ophthalmic diseases

### 3.1 Infective diseases

In 1928, Alexander Fleming made a seminal discovery that transformed medicine by observing that a *Penicillium* mold [later identified as *Penicillium rubens* Biourge, (Ascomycota)] inhibited the growth of *Staphylococcus* bacteria in contaminated cultures, leading to the identification of penicillin and the beginning of the antibiotic era (Houbraken et al., 2011; Gyéresi and Papp, 2022). Although *Penicillium* species are common environmental and food-associated molds [e.g., *P. roqueforti* Thom (Ascomycota) in cheese], their antibacterial properties had already been implicitly recognized, as mold was used in a primitive form in ancient Egypt for treating infections (Gould, 2016). Following its introduction, penicillin revolutionized the treatment of bacterial infections, significantly reducing mortality and becoming widely applied in ophthalmology for managing ocular infections and preventing vision loss. However, exposure through contaminated products or inappropriate use has contributed to antibiotic resistance and other health risks (Olson and Sanders, 1975; Gould, 2016). The introduction of penicillin revolutionized the treatment of bacterial infections, significantly reducing mortality rates. Its impact extended to ophthalmology, where it provided effective treatment for various ocular infections, thereby preserving vision and preventing blindness. Crystalline penicillin-G (averagely 18–24 IU/day) is administered for ocular syphilis, which is treated as neurosyphilis (Géhl et al., 2020) (Supplementary Table S1). This condition, known as the “chameleon of diseases,” can present with a wide range of ophthalmic symptoms, from anterior uveitis to chorioretinitis (Géhl et al., 2020). Fortunately, penicillin-resistant *Treponema pallidum* is still very rare (Géhl et al., 2020). However, it is crucial to focus on disease prevention in the future to avoid the development of widespread resistance.

In addition to penicillins, cephalosporins represent a major class of β-lactam antibiotics and constitute a cornerstone of modern antimicrobial therapy through inhibition of bacterial cell wall synthesis. These compounds were originally derived from *Acremonium chrysogenum* (Thirum. & Sukapure) W. Gams (Ascomycota) (Supplementary Table S1). In ophthalmology, their evidence-based use is extensive, and they are considered first-line agents in several clinical settings: for instance, intracameral cefuroxime injection is routinely administered at the conclusion of cataract surgery to significantly reduce the risk of postoperative endophthalmitis. A large prospective study conducted by the European Society of Cataract and Refractive Surgeons demonstrated that prophylactic intracameral cefuroxime reduced the incidence of post-cataract endophthalmitis from 2.96 to 0.62 cases per 1,000 surgeries (Wykoff et al., 2011). In the treatment of established infections, ceftazidime is widely used as an intravitreal agent due to its potent Gram-negative coverage, making it a key component in the management of suspected or confirmed bacterial endophthalmitis (Wykoff et al., 2011; Meyer et al., 2021).

Fusidic acid, isolated from *Fusidium coccineum* Corda (Ascomycota), is another fungal-derived antibiotic with established ophthalmic use (Supplementary Table S1). Topical formulations are indicated for the treatment of bacterial conjunctivitis, with activity primarily against Gram-positive organisms, particularly *Staphylococcus aureus*, as well as selected Gram-negative species such as *Haemophilus* spp. Clinical studies have shown that fusidic acid viscous eye drops administered twice daily are comparable in efficacy to tobramycin drops given four to six times daily, in terms of clinical resolution and bacteriological outcomes (Jackson et al., 2002). Adverse effects are generally mild and infrequent, including transient ocular irritation, burning, or tearing, with occasional localized hypersensitivity reactions.

Echinocandins (including caspofungin, micafungin, and anidulafungin) are antifungal agents derived from species such as *Aspergillus nidulans* (Eidam) G. Winter and *Glarea lozoyensis* Bills, Peláez & Polishook (Ascomycota), and are currently first-line systemic therapies for candidemia. However, their role in ophthalmology remains limited. Due to poor penetration across the blood–retinal barrier, echinocandins are generally not preferred for the treatment of established intraocular fungal infections, such as endogenous endophthalmitis or fungal keratitis. While off-label administration through topical or intravitreal routes has been explored, such approaches remain investigational, supported primarily by small-scale or exploratory studies rather than standardized clinical protocols (Patil and Majumdar, 2017; Hillenbrand et al., 2022).

Pleuromutilins, a class of tricyclic diterpenoid antibiotics first isolated from *Clitopilus* spp. [*Clitopilus scyphoides* (Fr.) P. Kumm. (Basidiomycota) and *Clitopilus passeckerianus* (Pilát) Singer (Basidiomycota)], have found clinical application mainly in dermatology and respiratory infections. Topical retapamulin (1%) is approved for the treatment of impetigo caused by methicillin-susceptible *S. aureus* and *Streptococcus pyogenes*. Although impetigo may involve the periocular region, no validated ophthalmic indications or clinical studies are currently available. Similarly, lefamulin, another pleuromutilin derivative, has been investigated for the treatment of community-acquired pneumonia, with no demonstrated relevance in ophthalmology to date (Goudarzi et al., 2021).

Overall, among fungal-derived antibiotics, only selected classes—most notably β-lactam antibiotics and fusidic acid—have well-established roles in ophthalmology supported by robust clinical evidence. In contrast, pleuromutilins lack validated ocular applications and remain confined to non-ophthalmic indications. Although echinocandins are increasingly used in systemic fungal infections with potential ocular involvement, their limited intraocular penetration and the lack of prospective clinical trials constrain their utility in targeted ophthalmic therapy. Thus, despite the historical and pharmacological importance of fungal-derived antimicrobials, their direct translation into evidence-based ophthalmic treatments remains relatively limited.

### 3.2 Dry eye disease

Dry eye disease is a multifactorial ocular surface disease characterized by altered tear film homeostasis, ocular symptoms, and neurosensory abnormalities affecting 5%–50% of the population (Berta et al., 2018; Tóth et al., 2021; Módis and Süveges, 2023). In recent years, there has been a notable increase in the number of conservative ophthalmic preparations containing natural active ingredients (Rák and Csutak, 2024b). Among these, medicinal mushroom-based active ingredients have gained significant attention. Anti-inflammatory and immunosuppressive agents can be effectively used to reduce ocular surface inflammation and increase tear production, which are the cause and effect of severe immune-mediated ocular surface inflammation and dry eye associated with Sjögren’s syndrome, systemic lupus erythematosus, systemic sclerosis, rheumatoid arthritis, and vernal keratoconjunctivitis (Yagci and Gurdal, 2014; Berta et al., 2018; Aragona et al., 2021; Rák and Csutak, 2024b). Eye drops containing 0.05% cyclosporin-A derived from *Tolypocladium inflatum* W. Gams (Ascomycota) inhibit ocular surface inflammation, migration of T-lymphocytes and apoptosis; however, they generally require a longer period to achieve therapeutic efficacy than corticosteroids (Supplementary Table S1) (Yagci and Gurdal, 2014; Aragona et al., 2021; Rák and Csutak, 2024b). Systemic side effects are not expected due to the low absorption potential across the ocular surface mucosa, and local unpleasant burning sensation may occur in some patients (Yagci and Gurdal, 2014; Rák and Csutak, 2024b). In addition to cyclosporine, another well-known DMARD (Disease-Modifying Antirheumatic Drug) therapy is mycophenolate mofetil, extracted from *Penicillium brevicompactum* Dierckx (Ascomycota), which is a prodrug of mycophenolic acid depletes guanosine triphosphate and suppresses the *de novo* synthesis of purines, thus inhibiting proliferation and inducing apoptosis of activated T-lymphocytes (Supplementary Table S1) (Feng et al., 2022). Mycophenolate mofetil is an effective immunosuppressive agent used in the treatment of moderate endocrine orbitopathy (Bartalena et al., 2021; Feng et al., 2022), ocular pemphigoid, autoimmune uveitis, scleritis, and ocular surface inflammations (Daniel et al., 2010). Side effects are rare but can include gastrointestinal issues (Daniel et al., 2010). In recent years, animal studies and clinical trials have been conducted on various mushroom-based active ingredients. One such example is *Isaria cicadae* Miq. (Ascomycota), an entomopathogenic fungus that parasitizes the larvae of *Cicada flammata* Dist. and is known for its immunomodulatory properties in TCM (Lin et al., 2017) (Supplementary Table S1). In a randomized, double-blind, placebo-controlled clinical trial conducted by Chang et al. (2022), supplementation with *I. cicadae* mycelium showed significant improvement in dry eye symptoms. Participants received daily doses of either 500 mg or 1,000 mg (Chang et al., 2022). The study results indicated that the treatment improved tear film osmolarity and increased TBUT (tear break-up time) values. However, there was no significant change in tear production based on the Schirmer test compared to the placebo group (Chang et al., 2022). This aligns with the understanding that dry eye disease is not merely a deficiency in tear production but involves inflammation and disruption of ocular surface homeostasis (Berta et al., 2018). Additionally, Lin et al. (2017) BAC-induced dry eye mouse model demonstrated that the mushroom extract reduces apoptosis of corneal epithelial cells and maintains conjunctival goblet cells, which helps alleviate dry eye symptoms (Lin et al., 2017; Chang et al., 2022). The treatment also reduced levels of inflammatory markers such as IL (interleukin)-1β and TNF (tumor necrosis factor)-α in the tear film (Chang et al., 2022), contributing to decreased inflammation and improved ocular surface health. These findings suggest that I*. cicadae* extracts could be a promising future ingredient for dietary supplements or artificial tears. Recent studies have investigated the potential of medicinal mushroom-based ingredients for the treatment of dry eye disease. Areesanan et al. (2025) examined six medicinal mushrooms: *Fomes fomentarius* (L.) Fr. (Basidiomycota), *G. lucidum*, *Ganoderma tsugae* Murrill (Basidiomycota), *Gloeophyllum odoratum* (Wulfen) Imazeki (Basidiomycota), *Laricifomes officinalis* (Vill.) Kotl. & Pouzar (Basidiomycota), and *Inonotus obliquus* (Fr.) Pilát (Basidiomycota), using *in vitro* cell cultures. According to their study, extracts of *G. lucidum* and *G. odoratum* exhibited significant antioxidant and anti-inflammatory effects, reducing reactive oxygen species (ROS) levels and the secretion of inflammatory cytokines in UVB-exposed human corneal epithelial cells and Meibomian gland cells (Supplementary Table S1). These extracts demonstrated low cytotoxicity, making them promising candidates for future dry eye treatments, even at low concentrations (Areesanan et al., 2025). According to TCM, *G. lucidum* does not exert a direct “moistening-dryness” effect. Instead, its therapeutic influence arises from restoring fundamental physiological substrates, Yin, Qi, and Blood, primarily through liver-kidney tonification and spleen strengthening with Qi replenishment. By reconstituting these systemic resources, *G. lucidum* indirectly nourishes the ocular orifices and thereby helps to alleviate dryness, irritation, and discomfort associated with dry eye disease (Zhu et al., 2021).

### 3.3 Cornea

Ocular surface diseases, including corneal ulcers, perforation, and blindness, are particularly prevalent in the African continent (Al-Ghadeer and Al-Amry, 2021; Okenwa-Vincent et al., 2023). Medicinal mushroom-based active ingredients offer promising opportunities for developing new conservative treatments. *G. lucidum* polysaccharides (GLP) have been shown to significantly reduce levels of inflammatory cytokines such as IL-1β and TNF-α, as well as the activity of the NF-κB (Nuclear Factor Kappa B) signaling pathway in corneal cells (Seweryn et al., 2021; Okenwa-Vincent et al., 2023). In a study by Okenwa-Vincent et al. (2023), rabbits with artificially induced corneal ulcers using sodium hydroxide and *Pseudomonas aeruginosa* infection showed significant clinical improvement when treated with aqueous extracts of *G. lucidum* compared to the placebo group. GLP treatment also demonstrated a 30% reduction in ROS levels in corneal cells, indicating its antioxidant properties. Additionally, GLP treatment increased corneal cell proliferation by 25% and accelerated wound healing (Okenwa-Vincent et al., 2023). These findings suggest that *G. lucidum* extracts could be a promising future ingredient for ocular surface disease treatments. Yan et al. (2015) were the first to present their research on the antiviral protein RC28, isolated from *Cortinarius caperatus* (Pers.) Fr. (Basidiomycota) and produced using an *Escherichia coli* vector. They tested the antiviral effects of this protein against herpes simplex virus-1 (HSV-1) (Supplementary Table S1). The cloned peptide delayed the occurrence of stromal keratitis and alleviated the severity of the disease, demonstrating strong antiviral activity in a mouse model of HSV-1 keratitis (Yan et al., 2015). These findings may support the future development of novel natural antiviral agents for the treatment of ocular herpes.

### 3.4 Glaucoma

Glaucoma is a chronic neurodegenerative spectrum disease, with progressive optic neuropathy as the common endpoint (Jonas et al., 2017; Kovács-Valasek et al., 2023; Pazos et al., 2025). Currently, it requires lifelong management and treatment, but it is not a curable condition (Jonas et al., 2017; Kovács-Valasek et al., 2023). Based on current knowledge, the only modifiable risk factor in its treatment has traditionally been the reduction of intraocular pressure (IOP). However, in recent years, molecular approaches targeting the vascular dysregulation, inflammatory cascade and apoptotic-autophagy pathways have gained prominence (Kovács-Valasek et al., 2023). Both endpoints are also targeted in therapeutic experiments involving medicinal mushrooms. Amen et al. (2017) tested 35 compounds from *Ganoderma lucidum* for Rho-kinase inhibitory effects (Supplementary Table S1). Their findings suggest that lanostane triterpenes may contribute, at least in part, to the observed ROCK(Rho-associated protein kinase)-I and ROCK-II inhibitory activity of *G. lucidum* (Amen et al., 2017; Rák and Csutak, 2024a). Synthetic Rho-kinase inhibitors have emerged as prospective therapeutic treatments for conditions such as glaucoma (Kovács-Valasek et al., 2023), therefore these findings could aid in the development of natural Rho-kinase inhibitors. The Xylariaceae family consists largely of tiny ascomycetous fungi, and their metabolites have antioxidant and anti-inflammatory properties (Lin et al., 2019; Rák and Csutak, 2024a). Lin et al. (2019) examined the retinoprotective compound Theissenolactone C (LC53), which is a fungal derivative extracted from *Theissenia cinerea* Y.M. Ju, J.D. Rogers & H.M. Hsieh (Ascomycota) (Supplementary Table S1). The study found that the application protected the retina by inhibiting NF-κB and MMP-9 (matrix metalloproteinase-9) activity following high-IOP-induced retinal damage (Lin et al., 2019; Rák and Csutak, 2024a). These findings suggest potential therapeutic applications for glaucoma and other retinal ischemia-related ocular disorders. The findings of both studies require further evaluation in human clinical trials to provide evidence-based information on the possibility of glaucoma complementary therapy (Rák and Csutak, 2024a). The study by Hong et al. (2021) evaluated the reduction of IOP in rats with glaucoma using *I. cicadae* mycelia extract (Supplementary Table S1). The extract showed significant IOP-lowering and antioxidant activities, making it a potential food supplement for relieving glaucoma symptoms (Hong et al., 2021). The study suggests that *I. cicadae* mycelia may represent a promising natural therapeutic resource for glaucoma treatment. Although *Wolfiporia extensa* (Peck) Ginns (Basidiomycota) and *Polyporus umbellatus* (Pers.) Fr. (Basidiomycota) are primarily incorporated into the herbal pharmacotherapy of anti-glaucoma products in TCM, their molecular effects on aquaporin channels and the downregulation of TGF-β1 (Transforming Growth Factor Beta 1) and Smad necessitate further clinical studies to determine their efficacy (Yang et al., 2018; Ou et al., 2020) (Supplementary Table S1).

The diagnostic and therapeutic advancements in glaucoma have been supported by continuous large population studies (Pazos et al., 2025). Therefore, in innovative therapeutic research for this disease group, it is essential to compare their efficacy with currently evidence-based therapies. Although the use of medicinal mushroom extracts is still in its infancy, there are very promising initial signs with *Pleurotus* spp. indicating a need for a more scientific approach in medicinal mushroom research (Supplementary Table S1). Ebigwai et al. (2012) investigated the effect of cold-water extract of *Pleurotus ostreatus* (Jacq.) Quél. (Basidiomycota) on increased IOP in a steroid-induced mouse glaucoma model. Their results showed that, compared to timolol eye drops, the extract produced dose-dependent IOP reductions: 44.78% at 100 mg/mL, 57.69% at 150 mg/mL, and 70.03% at 200 mg/mL (Ebigwai et al., 2012). Odjimogho et al. (2024) documented significant IOP-lowering effects and antioxidant activity comparable to ascorbic acid in a steroid-induced rabbit glaucoma model with *Pleurotus tuber-regium* (Rumph. ex Fr.) Singer (Basidiomycota) at doses of 200 and 400 mg/kg. Their study highlighted that the pharmacological effects of the mushroom extract were similar to those of D-mannitol and acetazolamide treatments (Odjimogho et al., 2024). Akinlabi and Erhabor (2016) compared the extract of *P. tuber-regium* with latanoprost 0.005% eye drops in a steroid-induced cat glaucoma model. Both *P. tuber-regium* and latanoprost significantly reduced dexamethasone-induced IOP elevation. They observed that the mushroom’s effect was more effective during the light cycle, similar to beta-blocker eye drops (as seen in Ebigwai et al.'s experiment in 2012), which are ineffective at night, while latanoprost is known to be effective during the dark cycle, with no significant difference in IOP-lowering efficacy (Akinlabi and Erhabor, 2016).

Innovative solutions are needed not only in conservative glaucoma treatment but also in surgical procedures, where the role of medicinal mushrooms is underrepresented but not impossible. Batterbury et al. (2002) conducted an *in vitro* experiment to confirm the anti-fibrotic effect of lectins derived from *Agaricus bisporus* (J. E. Lange) Inbach (Basidiomycota) on human Tenon-capsule fibroblasts (Supplementary Table S1). The *A. bisporus* lectin caused a dose-dependent inhibition of proliferation and lattice contraction without significant toxicity. Proliferation was inhibited by 5%–40% in the dose range 0.02–0.1 mg/mL, but complete inhibition was achieved with 0.1 mg/mL, similarly to standard Mitomycin-C solution (Batterbury et al., 2002; Muszyńska et al., 2017). The experiment has not been confirmed on animals since then. In the Far East, the red-colored *Monascus purpureus* Went (Ascomycota) is used as a food coloring agent (e.g., for Peking duck, sweets, etc.), but the lovastatin extracted from it inhibits cholesterol synthesis (Park et al., 2016). However, Park et al. (2016) aimed to investigate the effect of lovastatin on wound healing as a natural antifibrotic agent (0.1 mg/mL) following trabeculectomy in New Zealand white rabbits (Supplementary Table S1). The efficacy was also compared with 0.2 mg/mL Mitomycin-C and balanced salt solution control groups. Their results showed that lovastatin significantly prolonged bleb survival compared to the control group, equivalent to Mitomycin-C, and similarly reduced the expected postoperative intraocular pressure (Park et al., 2016). The anti-inflammatory and antifibrotic effects were also successfully confirmed histologically (Park et al., 2016). Following the previous examples, it is noteworthy that there are few studies on oral antifibrotic agents, as they are typically applied locally in filtration surgeries. However, Hu et al. (2024) demonstrated the effect of the Qingguang’an capsule containing 20 g of *W. extensa* in a rabbit model of trabeculectomy (Supplementary Table S1). Their experiment showed that administering the capsule orally twice daily for 15 days objectively reduced autophagic biochemical pathways in the Tenon capsule following trabeculectomy. Compared to animals treated with 0.4 mg/mL Mitomycin C solution, PBS (Phosphate-Buffered Saline) solution, and those without antifibrotic treatment, their observations indicated better bleb morphology and less neovascularization (Hu et al., 2024). Based on the results above, there is hope for the future development of new natural-based antifibrotic agents with fewer side effects.

### 3.5 Optic nerve trauma and inflammation

Optic nerve trauma initiates a cascade of inflammatory responses involving glial activation, blood-retina barrier disruption, and immune-cell recruitment, which collectively drive secondary neurodegeneration and contribute substantially to retinal ganglion cell loss (Xu et al., 2025). *Isaria sinclairii* (Berk.) Lloyd (Ascomycota), the sibling species of the previously mentioned *I. cicadae*, is used to produce the oral immunomodulatory drug fingolimod (Supplementary Table S1). It has been approved for the treatment of relapsing forms of multiple sclerosis, a condition that often begins with optic neuritis as an ophthalmic symptom. However, the drug is also known to have an ophthalmic side effect called fingolimod-associated macular edema (FAME) (Wang et al., 2022).

Erinacine A, a natural compound isolated from *H. erinaceus* (Bull.) Persoon (Basidiomycota), has been investigated for its anti-inflammatory and neuroprotective potential in several cell and animal models of Alzheimer’s disease, Parkinson’s disease, and traumatic nerve injuries (Hsu et al., 2023) (Supplementary Table S1). Hsu et al. (2023) conducted a randomized placebo-controlled study on a rat model with traumatic optic nerve injury to evaluate the neuroprotective effects of this medicinal mushroom on induced traumatic optic neuropathy. In groups treated with single and double doses of erinacine A, the flash Visual Evoked Potentials (VEP) P1–N2 amplitudes were 1.8 and 2.4 times higher, respectively, compared to the PBS control group. Compared with controls, retinal ganglion cell density was 2.3-fold and 3.7-fold higher, whereas the number of apoptotic retinal ganglion cells was reduced by 10.0-fold and 15.6-fold. Macrophage counts were also reduced by 1.8-fold and 2.2-fold in the single- and double-dose groups, respectively. Treatment with single and double doses of *Hericium erinaceus* extract reduced the levels of inflammatory and oxidative stress markers detectable by Western blot [phosphor-receptor-interacting serine/threonine-protein kinase 1 (pRIP1), caspase 8 (Cas8), cleaved caspase 3 (cCas3), TNF-α, TNF-receptor1 (TNFR1), IL-1β, inducible nitric oxide synthase (iNOS)], while increasing the levels of antioxidant enzymes [nuclear factor erythroid 2-related factor 2 (Nrf2), hem oxygenase-1 (HO-1), and superoxide dismutase 1 (SOD1)] compared to the control group (Hsu et al., 2023). Given the shared mechanisms of neurodegeneration, these findings may warrant further investigation in glaucoma models; however, such applications have not yet been studied.

### 3.6 Cataract

Cataract is a major ocular problem (from the Ancient Greek καταρράχτης—“rapid waterfall”) defined by the opacification or loss of transparency of the crystalline lens in the eye (Isai et al., 2009; Tewari et al., 2019). Prevalence of cataract increases with age (as a non-modifiable risk factor), thus in our progressively aging population it reaches 64% among individuals over 70 years (Tewari et al., 2019). In addition to aging, traumatic etiology, especially in young individuals, can also lead to the development of cataracts. Interestingly, in the context of fungi, Nath et al. (2022) report a case study of a 23-year-old male patient who experienced blunt eye trauma and subsequently developed traumatic cataracts in his lens resembling *Flammulina filiformis* Z. W. Ge, X. B. Liu and Zhu L. Yang (Basidiomycota) (Nath et al., 2022) (Supplementary Table S1). Surgery is the only way to correct cataracts and recover visual refraction in evidence-based integrative medicine, however, the invasiveness, cost and environmental impact of cataract surgery may discourage some individuals; moreover, postsurgical infection may impair recovery of effective vision (Isai et al., 2009; Cicinelli et al., 2023). Thus, the development of ethnopharmacological approaches capable of preventing or delaying cataract formation would be of considerable interest in integrative medicine, however, currently there is no evident alternative method (Isai et al., 2009). Isai et al. (2009) reported anticataractogenic effect of *P. ostreatus* (Jacq.) Quél. in an experimental *in vivo* selenite-induced cataract rat model. 300 mg/kg extract could delay the cataractogenesis in 75% of the experimental rodents (Isai et al., 2009) (Supplementary Table S1). *In vitro*, the simultaneous incubation of the fungal extract and selenite reduced lens opacity by maintaining antioxidant components at near-normal levels (Isai et al., 2009). Cai et al. (2019) isolated asperpyrone F, dimer naphthol-γ-pyrone with antioxidant and radical scavenging properties (Cai et al., 2019; Zhao Q. et al., 2024), which may contribute to the observed anticataractogenic effects; however, this hypothesis has not yet been directly confirmed experimentally. Ganeshpurkar et al. (2011) evaluated the *in vitro* effects of *P. ostreatus* var. florida Cetto (Basidiomycota) extract on goat lenses with hyperglycemic aqueous humor incubation-induced cataracts. The *Pleurotus florida* extract at concentrations of 250 μg/mL and 500 μg/mL significantly reduced malondialdehyde levels and increased catalase and glutathione levels, which are antioxidant enzymes important for preventing oxidative damage to the lens, thereby preserving lens transparency (Ganeshpurkar et al., 2011). Lu et al. (2023) demonstrated that *I. cicadae* mycelial extracts, containing the bioactive compound N6-(2-hydroxyethyl) adenosine, were investigated for their effects on cataract formation in a UVB-induced mouse model. They found significant reduction in malondialdehyde levels and an increase in the activity of antioxidant enzymes such as superoxide dismutase (SOD) and catalase. The extracts also decreased the expression of heat-shock proteins (HSPs), which are associated with cataract formation (Lu et al., 2023). They found that the *I. cicadae* significantly reduced oxidative stress and inflammation in the lens epithelial cells. By adding two additional components to the Bā Wèi Dì Huáng Wán formula (containing 3 g of *W. extensa*) in Kampo medicine, Isobe et al. (2003) tested it on healthy adult volunteers. Their results indicated that the systolic, diastolic, and mean velocities of the central retinal artery were significantly higher compared to other single herbal components (e.g., *Cinnamomum cassia* (L.) J.Presl). Previous rodent studies demonstrated that the Bā Wèi Dì Huáng Wán formula successfully delayed senile cataract (Isobe et al., 2003).

### 3.7 Diseases affecting the macula

The number of patients suffering from age-related macular degeneration (AMD) is increasing as society ages (Pámer, 2013; Kovács-Valasek et al., 2023). For the conservative treatment of dry AMD, the AREDS2 study has provided a globally recognized combination of dietary supplement ingredients (Pámer, 2013; Kovács-Valasek et al., 2023). Interestingly, in Italy, there is an eye vitamin product available (Macuprev®, Farmaplus Italia s.r.l., Italy) that includes the AREDS2 formula along with additional active ingredients, including a medicinal mushroom extract. The composition of Macuprev® includes lutein, zeaxanthin, N-acetylcysteine, bromelain, vitamin D, vitamin B12, alpha-lipoic acid, rutin, vitamin C, zinc oxide, *Vaccinium myrtillus* L. (Ericaceae), and *G. lucidum* (Parravano et al., 2019). Parravano et al. (2019) conducted a double-blind, placebo-controlled, randomized, prospective study to evaluate the effects of daily supplementation with two Macuprev® tablets in 30 patients with intermediate AMD over 6 months. Their results showed a significant increase in the multifocal electroretinogram (mfERG) response amplitude density in the central macular areas for those consuming the active ingredient tablets (Supplementary Table S1). However, there were no significant structural changes in the retina and choroid SD-OCT parameters (Parravano et al., 2019). Thus, Macuprev® supplementation enhances the function of the macular pre-ganglionic elements without causing significant ultrastructural changes. The placebo-controlled mouse model study by Ostrovski and Zinner (2024) investigates the potential of *Grifola frondosa* (Dicks.) Gray (Basidiomycota) in treating AMD due to its high ergothioneine content (Supplementary Table S1). Ergothioneine is a potent antioxidant derived from histidine (Muszyńska et al., 2017; Ostrovski and Zinner, 2024). Physiologically, it is found in high concentrations in red blood cells, semen, skin, and notably in the lens of the eye (Muszyńska et al., 2017), where it functions as part of the endogenous antioxidant redox system. This study proposes testing therapeutic ergothioneine in mice, administering 2 mg/10 mL/kg daily for 4 weeks, with melatonin (10 mg/10 mL/kg) as a control (Ostrovski and Zinner, 2024). Quantitative techniques showed that the measured biomarkers of oxidative stress (lipofuscin, 8-oxoguanine, mitochondrial DNA damage) were reduced, thereby decreasing AMD progression and supporting its potential as a therapeutic agent (Ostrovski and Zinner, 2024). The study integrates TCM with Western research and suggests that *G. frondosa* could be a novel therapeutic option for AMD. Current reports by Wang and Cunnusamy (2012) and Wang and Cunnusamy (2013) evaluate a new alternative therapeutic composition for wet AMD developed by [Jin (2012); patent No: WO/2012/079419]. According to their description, the formula can enhance and stabilize the vision of AMD patients, promote the absorption of macular exudate, edema, and hemorrhage, and reduce CNV (choroidal neovascularization) leakage and the CNV area in the macular region. However, the role of *W. extensa* in AMD is not clearly or adequately explained in this report. Li et al. (2022) compiled TCM formulas relevant to AMD. They identified 2 formulas for dry AMD, 5 formulas for wet AMD, and 6 formulas for unspecified AMD that included *W*. *extensa*. Additionally, *P. umbellatus* was exclusively used in 3 formulas for wet AMD. Although there are not many studies on the effectiveness of single *W. extensa* in the treatment of AMD, according to Li et al. (2022) a large dose of *W. extensa* combined with Jiangtang Huoxue formula has been shown to reduce macular exudation area, the number of microhemangiomas in retinopathy patients, and the serum VEGF (vascular endothelial growth factor) level. They suggest that these mushrooms are potent VEGF inhibitors reducing the number and permeability of microhemangiomas (Li et al., 2022).

Diabetic retinopathy often leads to diabetic macular edema due to the disruption of the blood-retinal barrier and altered hemodynamics, causing extracellular fluid accumulation (Huai et al., 2023; Rák et al., 2023). As one of the most common clinical complications of diabetes mellitus, it has become the leading cause of blindness in middle-aged and older adults (Huai et al., 2023; Rák et al., 2023). Huai et al. (2023) report a computer search of the Chinese Journal Full Text Database and Wanfang database for clinical cases related to the treatment of macular edema, ranking *W. extensa* first with a frequency of 60 recurrences, suggesting that the TCM community generally recognizes the efficacy of *W. extensa* in the treatment of diabetic macular edema. However, its specific mechanism of action needs to be further explored.

Retinal vein occlusion is the second most common retinal vascular disease after diabetic retinopathy, with a prevalence of 0.72%. Its most serious complication is macular edema, that can lead to irreversible visual impairment and occurs in 5%–15% of patients with branch retinal vein occlusion within 1 year of onset (Su et al., 2022). Su et al. (2022) concluded that the current data on the combination of TCM herbal medicines and anti-VEGF injections are promising but inconclusive. They may be more beneficial to retinal vein occlusion-associated macular edema than anti-VEGF alone (Su et al., 2022), however, due to the limitation of sample size and methodological quality, a large sample size and multicenter well-designed clinical study are required.

However, there are relatively few TCM records on the use of *W. extensa* as a treatment for central serous chorioretinopathy, so any connection of its effectiveness is speculative at this point.

Although griseofulvin, a fungitoxic agent originally isolated from *Penicillium griseofulvum* Dierckx (Ascomycota), has been widely used in clinical practice for the treatment of dermatophyte infections, recent preclinical studies have highlighted its potential in inhibiting CNV (Supplementary Table S1). This effect appears to be mediated by off-target inhibition of ferrochelatase, a key enzyme in heme biosynthesis. Mechanistically, griseofulvin is metabolized into N-methyl-protoporphyrin IX, a competitive inhibitor of ferrochelatase. This inhibition disrupts heme production and subsequently impairs endothelial cell proliferation, migration, and angiogenic activity, all of which are critical processes underlying CNV formation. Despite robust anti-angiogenic effects demonstrated *in vitro* and in animal models, clinical translation (e.g., in the treatment of wet-AMD, diabetic retinopathy or retinal vein occlusion, etc.), remains limited. Current evidence is confined to preclinical systems, and several key aspects - including ocular pharmacokinetics, intraocular bioactivation, and long-term safety - require further investigation. Nevertheless, given its FDA-approved status and well-characterized mechanism of action, griseofulvin represents a promising candidate for drug repurposing, warranting further translational and early-phase clinical studies (Corson et al., 2016; 2023; Chobisa et al., 2023).

### 3.8 Vitreoretinal diseases

Proliferative vitreoretinopathy is characterized by the formation of scar-like membranes on the retinal surfaces and is seen in around 10% of rhegmatogenous retinal detachments. Retinal pigment epithelial (RPE) cells are pivotal in the development of proliferative vitreoretinopathy and although the exact mechanism is unknown, RPE cells become detached from Bruch’s membrane and assume a wound repair phenotype similar to wound fibroblasts or macrophages (Kent et al., 2003a; Kent et al., 2003b; Muszyńska et al., 2017). Kent et al. (2003a) and Kent et al. (2003b) aimed to investigate whether the lectin of *A. bisporus* can bind to human RPE and affect their behavior, specifically in the context of proliferative vitreoretinopathy. *A. bisporus* lectin specifically binds Thomsen Friedenreich (TF) antigen, which is present in RPE cells and their extracellular matrix (Kent et al., 2003a; Kent et al., 2003b; Muszyńska et al., 2017) (Supplementary Table S1). In their experiment, the lectin showed higher affinity to altered RPE or extracellular matrix components, especially to proliferative epiretinal membranes, indicating a glycan-rich environment and potential therapeutic targeting (Kent et al., 2003a; Kent et al., 2003b; Muszyńska et al., 2017). Furthermore, the lectin significantly inhibits collagen matrix contraction in a concentration-dependent manner, suggesting it may reduce fibrotic changes. Fortunately, no cytotoxicity was detected at the tested concentrations, supporting its safety as a potential treatment approach (Kent et al., 2003a; Kent et al., 2003b; Muszyńska et al., 2017). According to TCM dietary recommendations, “Double mushroom soup” (双耳汤) is suggested for vitreous hemorrhage. It contains *A. heimuer* F. Wu, B. K. Cui, Y. C. Dai (Basidiomycota) and *Tremella fuciformis* Berk. (Basidiomycota) mushrooms (Supplementary Table S1). The scientific rationale associated with the vitreous body is based on the report of Chandra et al. (2022) and Kalitukha et al. (2023). These reviews report that *T. fuciformis* and *Auricularia heimuer* polysaccharides are ideal as a drug vehicle for effective ocular drug delivery due to their similar structure to hyaluronic acid, non-toxic nature, easy solubility in water, and good colloidal properties and stability. *T. fuciformis* eye gel mask is used as a skincare product due to its moisturizing effect, which has effects similar to or even superior to hyaluronic acid (Colomer et al., 2016), and A*. heimuer* also has ideal characteristics to become a cosmetic ingredient (Kalitukha et al., 2023). The authors could not find relevant scientific data on both fungi and vitreous hemorrhage directly, but ethnopharmacology studies indicate its use in abnormal clotting (Colomer et al., 2016). However, a preclinical study investigates the impact of *T. fuciformis* polysaccharides on early brain injury following subarachnoid hemorrhage, which is not a direct analogy to vitreous hemorrhage (Wan et al., 2025). The study utilized both *in vivo* (animal models) and *in vitro* (cell cultures) approaches to assess the effects of mushroom polysaccharides on microglial activity and neuronal apoptosis post-subarachnoid hemorrhage (Wan et al., 2025). Key findings suggest that *T. fuciformis* polysaccharide administration reduced cerebral edema and decreased neuronal apoptosis by inhibiting the KDR-mediated P38 MAPK/NF-κB pathway (Wan et al., 2025). Further research and validation are necessary to confirm these findings and fully understand the therapeutic potential of *T. fuciformis* polysaccharide in the context of subarachnoid hemorrhage. Su et al. (2022) and Huai et al. (2023) mention that according to the TCM, *W. extensa* “nourishes and harmonizes blood,” and “removes blood stasis” which ameliorates intraocular hemostasis and hemorrhage (Su et al., 2022), suggesting a complementary therapy in vitreous hemorrhage.

## 4 Discussion

Although the medical use of various medicinal mushrooms has been documented for 5,000 years in human history (Veilleux et al., 2018; Rák and Csutak, 2024a), the scientific validation of phytotherapy and mycotherapy is still ongoing. The pharmacological effects of various culinary and medicinal mushrooms are discussed in mycotherapy (Rák and Csutak, 2024a), which is legally treated within the field of phytotherapy in certain countries, such as Hungary, despite the fact that mushrooms do not belong to the kingdom *Plantae*. Within this field, a specialized branch focuses on the therapeutic effects of *G. lucidum*, known as ganotherapy. The therapeutic potential of medicinal mushrooms is largely attributed to their bioactive compounds. These include polysaccharides, triterpenoids, ergosterols, and various antioxidants (Rák and Csutak, 2024a). Polysaccharides like β-glucans are known for their immune-modulating properties, while triterpenoids possess anti-inflammatory and antioxidant effects (Rák and Csutak, 2024a). Importantly, only a limited subset of mushroom-derived compounds has reached higher levels of clinical evidence (Level 1–2), primarily represented by antimicrobial and immunomodulatory agents already integrated into conventional pharmacotherapy. These include β-lactam antibiotics such as penicillin derived from *P. rubens* and cephalosporins from *A. chrysogenum*, as well as fusidic acid from *F. coccineum*, which are routinely used in the management of ocular infections. In addition, immunomodulatory agents of fungal origin, including cyclosporine A from *T. inflatum* and mycophenolate mofetil derived from *P. brevicompactum*, have well-established roles in the treatment of immune-mediated ocular diseases such as dry eye associated with autoimmune conditions, uveitis, and ocular surface inflammation. Furthermore, emerging clinical evidence supports the use of *I. cicadae* in dry eye disease (Level 2), while *G. lucidum* has demonstrated moderate clinical evidence in AMD, particularly as part of combination therapies. In contrast, the majority of medicinal mushrooms discussed in this review - including species such as *Pleurotus* spp., *H. erinaceus*, *W. extensa*, and *P. umbellatus* - remain confined to preclinical or traditional use contexts (Level 5 evidence). Their proposed ophthalmic benefits are largely supported by mechanistic studies, *in vitro* and *in vivo* models, and ethnopharmacological data, rather than robust clinical validation. This disparity underscores a substantial translational gap between traditional knowledge and evidence-based ophthalmic therapeutics.

In TCM ophthalmology, two medicinal mushrooms are particularly important and are discussed in detail in the following sections. Among these, the sclerotia of *W. extensa* (Peck) Ginns (Basidiomycota) is commonly included as a principal component of TCM ophthalmic prescriptions. Approximately 10% of the formulations listed in the Pharmacopoeia of the People’s Republic of China contain *W. extensa*, primarily because of its recognized diuretic properties. The hardened mycelia are parasites on the roots of *Pinus massoniana* Lamb. (Pinaceae) and *Pinus densiflora* Siebold & Zucc. (Pinaceae) trees in China, Japan, Korea and North America (Guo et al., 2024). This particular medicinal formulation, which contains both the roots of pine trees and the sclerotium of the mushroom, is known as *Poria cum pini radice*. In TCM, it is used not only for reducing edema but also for its calming and sleep-enhancing effects (Yang et al., 2021). However, scientific literature has so far only reported its antiarrhythmic effects tested on a zebrafish model (Yang et al., 2021). The ethanol extract of *W. extensa* can inhibit the secretion of inflammatory mediators TNF-α, NO, PGE2 and IL-1β. *W. extensa* targets the response of macrophages via the inactivation of the NF-κB signaling pathway. The mushroom exerts its effects through the TGF-β1/Smad pathway as well, which is involved in edema reduction. Compared with furosemide, the aqueous extract of *W. extensa* appears to exert a potassium-sparing diuretic effect by lowering plasma arginine vasopressin levels and reducing vasopressin type 2 receptor mRNA expression (Guo et al., 2024). One example, in their review, Yarnell and Abascal highlight that the Liù Wèi Dì Huáng Wán formula, which includes 9 g *W. extensa*, is one of the most significant TCM formulations. It is used in the treatment of patients with glaucoma and cataracts due to its diuretic properties, although this application is more scientifically justified for glaucoma according to Western pharmacology (Yarnell and Abascal, 2013).

Another important medicinal mushroom in TCM is *P. umbellatus* (Pers.) Fr. (Basidiomycota), which has been included in numerous preparations listed in the 2020 Chinese Pharmacopoeia (Liu et al., 2021). In TCM clinical practice, the diuretic effect is mainly emphasized. Based on expert consensus, *P. umbellatus* is also part of the TCM glaucoma protocol in case of “Blood stasis and fluid retention syndrome” (Yang et al., 2018; Liu et al., 2021). A clinical example: Berlin’s edema, a transient retinal edema following blunt eye trauma, is treated in TCM with herbal preparations that include *W. extensa* and *P. umbellatus*. Although there are no modern scientific references specifically supporting this use, the speculative diuretic properties of these popular medicinal mushrooms may help explain its effectiveness. For chronic anterior uveitis, TCM dietary recommendations suggest the consumption of “Chrysanthemum and Winter Melon Soup” (菊花冬瓜汤), which includes 25 g of *Lentinula edodes* (Berk.) Pegler (Basidiomycota) as one of its ingredients. Pharmacologically, it contains immunomodulating polysaccharides (β-glucans) and antioxidant and anti-inflammatory phenolic compounds (syringic acid and vanillic acid) (Dai et al., 2023), which could justify its use. However, the authors did not find scientific descriptions supporting this in the literature.

### 4.1 Safety, toxicity and controversies

Historical and ethnopharmacological records indicate that mushroom-derived ocular applications have been used across diverse cultural contexts. Early sources [e.g., The Canon of Medicine by Ibn Sina (Avicenna) around 1000 AD] describe the use of truffle extracts for ocular conditions (Khalifa et al., 2019), and desert truffles, including *Terfezia claveryi* Chatin, *Terfezia boudieri* Chatin, and *Tirmania nivea* (Desf.) Trappe (Ascomycota), have been traditionally applied in the Middle East and sub-Saharan Africa (El Enshasy et al., 2013). Similar practices have been documented in West Africa, in Côte d'Ivoire, specifically in the Gagnoa and Soubré regions, where *Psathyrella tuberculata* (Pat.) A. H. Sm. (Basidiomycota) is applied directly to the ocular surface after mechanical maceration (N’Douba et al., 2022), and in Southeast Asia, where extracts or condensed water from *Cookeina* spp., including *Cookeina tricholoma* (Mont.) Kuntze and *Cookeina speciosa* (Fr.) Dennis (Ascomycota), are instilled into the eyes in ethnomedical contexts (Yusran et al., 2024). Ethnomycology researchers have also recorded similar eye-drop uses in other regions of Sulawesi. Despite the long-standing traditional use of such practices, specific ophthalmic safety data remain lacking. Notably, there are no published clinical reports directly linking non-sterile mushroom extracts to keratitis. However, the absence of documented adverse events should not be interpreted as evidence of safety. Evidence from ophthalmology demonstrates that non-sterile ophthalmic preparations can result in severe microbial keratitis, and exposure of the cornea to environmental organic materials is a well-established risk factor for fungal infections (Donovan et al., 2021). Given that mushrooms are complex biological substrates capable of harboring diverse bacterial and fungal communities, their direct, non-sterile application to the ocular surface represents a plausible and clinically relevant risk for both fungal and bacterial keratitis. Accordingly, the use of unsterilized mushroom-derived preparations as eye drops should be strongly discouraged due to the potential risk of sight-threatening infection.

Allergic reactions may occur with natural products, including medicinal mushrooms, and healthcare professionals involved in mycotherapy should be prepared to recognize and manage such reactions. The most well-known fungal compound allergies are β-lactam antibiotics (penicillin and cephalosporins), with approximately 10% of the population reporting them, although less than 1% are truly allergic (American College of Allergy Asthma and Immunology, 2010; Wykoff et al., 2011). Notably, approximately 80% of patients with IgE-mediated penicillin allergy lose their sensitivity after 10 years (American College of Allergy Asthma and Immunology, 2010). In contrast, cephalosporins with modified, more complex side chains, particularly second-(e.g., cefuroxime, cefprozil) and third-generation (e.g., ceftazidime, cefpodoxime) agents are associated with minimal risk of cross-reactivity (Wykoff et al., 2011). However, *L. edodes* mushroom may induce allergic reactions and a dermatological disorder called toxicodermia or shiitake dermatitis. The latter occurs 24–72 h after eating raw shiitake, whereas allergic reactions such as asthma, contact dermatitis or alveolitis develop after contact with the mushroom or its spores (Lippert et al., 2003; Tze et al., 2025). Lentinan, a thermolabile polysaccharide found in raw or undercooked shiitake mushrooms, has been implicated as the causative agent in shiitake mushroom dermatitis (Tze et al., 2025). Shiitake dermatitis can manifest with the following symptoms: itchy erythematous papules, often appearing in a linear pattern on the trunk, limbs, and sometimes the face; severe pruritus; vesicular eruptions; eczematous reactions; and flagellate dermatitis (Lippert et al., 2003; Tze et al., 2025). It is also important to note from an ophthalmic perspective that conjunctivitis and eyelid edema may occur (Lippert et al., 2003; Tze et al., 2025), although their exact prevalence is unknown. Perennial conjunctivitis or occupational rhinoconjunctivitis, such as in the food industry, are commonly known reactions to fungal spores (Endre, 2014). Regarding safety and allergenicity, a clear distinction must be made between microscopic fungal derivatives (such as beta-lactam antibiotics) and macrofungal preparations. In the context of medicinal mushrooms, the primary allergiological concern involves hypersensitivity to fungal proteins, high-molecular-weight polysaccharides, and airborne spores. Airborne macromushroom spores are well-known triggers for respiratory allergies and allergic conjunctivitis. Therefore, topical ophthalmic application of poorly purified mushroom extracts poses a potential risk of localized IgE-mediated ocular hypersensitivity, contact dermatitis of the eyelids, or severe ocular surface inflammation in sensitized individuals. Rigorous protein-depletion and purification protocols are mandatory for future ophthalmic formulations to mitigate these specific allergenic risks.

In a previous chapter, the authors mentioned in detail that the substitution of similar mushroom species or the lack of mushroom knowledge can lead to potentially fatal poisonings. Just as herbs can absorb contaminants and heavy metals from the environment (Rák and Csutak, 2024a), mushrooms can do the same. When sourcing mushrooms from their natural habitat or using them for medicinal or culinary purposes, one must consider the possibility of contamination. Among the medicinal mushrooms previously mentioned in our article, the *Cortinarius* spp. has been documented worldwide to accumulate extremely high levels of pre- and post-Chernobyl radioactive ^137^Cs (radiocaesium) (Zalewska et al., 2016).

Recently, iris-color–changing eye drops (e.g., Fancy Eye Drops™, LightEyez Ltd., United Kingdom) sold online have gained attention, including formulations advertised as containing *G. lucidum* extract. Although marketed as a non-invasive method to alter eye color, no pharmacological or clinical evidence supports that *G. lucidum*, or any mushroom-derived ingredient, can modify iris melanin or change eye color. The proposed mechanism remains entirely unsubstantiated. Moreover, the safety of such unregulated products is highly questionable. Major ophthalmic authorities (e.g., American Academy of Ophthalmology - AAO, U.S. Food and Drug Administration - FDA) emphasize that no color-changing eye drops has been approved, regardless of whether they contain mushroom extracts (AAO, 2024; Hutton, 2024). Potential risks include ocular inflammation, infection (due to microbial contamination in unregulated manufacturing), light sensitivity from altered pigment levels, and in severe cases irreversible corneal or retinal damage, which may lead to permanent vision loss (Moazed, 2020; Iskandar et al., 2022; AAO, 2024; Hutton, 2024). Thus, while mushroom-based compounds like *G. lucidum* continue to be investigated for therapeutic potential, their use in iris-color modification lacks scientific basis and poses significant safety concerns. Rigorous research and regulatory oversight are essential before medicinal mushroom-derived ingredients can be considered safe for ophthalmic use.

### 4.2 Limitations

While the potential benefits of medicinal mushrooms in ophthalmology are promising, there are several challenges and controversies that need to be addressed. Firstly, standardization remains a major limitation, as variability in species, cultivation conditions, and extraction methods leads to inconsistent biological activity and therapeutic outcomes. Secondly, the safety and efficacy must be rigorously validated in well-designed clinical trials. Although preclinical studies report promising findings, robust clinical evidence is still lacking, particularly regarding dose–response relationships. In particular, the existing literature suffers from a profound lack of dose-response studies. Without rigorous dose-escalation data, it remains impossible to determine the optimal therapeutic window, the minimum effective dose, or the threshold for ocular toxicity. Furthermore, preclinical studies rarely include direct comparisons with established reference therapies (e.g., commercial anti-inflammatory or anti-glaucoma eye drops), limiting the ability to assess relative efficacy. Methodologically, current *in vivo* evidence relies almost exclusively on acute animal models. These models fail to capture the chronic and progressive nature of major human ocular diseases (e.g., glaucoma, AMD, or diabetic retinopathy), thereby limiting translational relevance. Crucially, ocular pharmacokinetic data are entirely lacking. The penetration, distribution, and clearance of mushroom-derived compounds within ocular tissues, particularly in the posterior segment, remain unknown. Lastly, regulatory inconsistency further complicates clinical translation, as the absence of harmonized standards affects product quality, safety, and reproducibility. Addressing these methodological, pharmacological, and regulatory challenges is crucial for the successful integration of medicinal mushrooms into ophthalmology. Another limitation of this review is the inability to perform a formal meta-analysis. The available literature is highly heterogeneous with respect to species, extraction methods, bioactive profiles, experimental models, and outcome measures, precluding quantitative synthesis, and highlights the urgent need for standardized experimental designs and well-powered clinical trials in the field.

### 4.3 Future directions

Future research on medicinal mushrooms in ophthalmology should focus on the standardization of extracts, the clarification of molecular mechanisms, and the development of clinically relevant formulations. Although several species show promising antioxidant, anti-inflammatory, neuroprotective or antifibrotic effects in preclinical models, consistent quality control and reproducible bioactive profiles are essential before translation into clinical practice. To achieve this, a critical priority action must be the rigorous chemical and biological standardization of specific candidate extracts, most notably *G. lucidum*, to ensure batch-to-batch consistency in active triterpenoids and polysaccharides. Well-designed human trials are particularly needed in dry eye disease, glaucoma, corneal wound healing, and retinal disorders, where early experimental data suggest potential benefit. Specifically, immediate efforts should be directed toward initiating human clinical trials that evaluate the efficacy of topical formulations for dry eye disease, as well as their IOP-lowering capabilities in glaucoma patients. Furthermore, alongside clinical evaluation, performing robust corneal penetration and ocular bioavailability studies will be essential to determine whether these bioactive compounds can effectively traverse the ocular barriers. Integrating traditional applications - especially those from TCM - with modern pharmacological validation may help identify the most relevant species and formulations. Safety assessment, including allergenicity, toxicology and contamination risks, must remain a priority to ensure clinical applicability. Overall, medicinal mushrooms represent a promising, yet still under-explored, adjunctive therapeutic avenue in ophthalmology, requiring coordinated multidisciplinary research to move from empirical use to evidence-based integration.

## 5 Conclusion

The kingdom of *Fungi*, whether edible or poisonous species, is culturally, religiously, and historically linked to eye and ocular diseases. Medicinal mushrooms hold significant potential in the field of ophthalmology. While challenges remain, continued research and clinical validation could pave the way for their integration into modern eye care practices. Whether considered traditional remedies or emerging therapeutic candidates, medicinal mushrooms represent a promising area of research in ophthalmology. Future research on medicinal mushrooms in ophthalmology appears promising, with several avenues for further research. More well-designed clinical trials are needed to validate the therapeutic claims of medicinal mushrooms. Understanding the precise mechanisms of action of mushroom-derived compounds will aid in developing targeted therapies. Exploring the synergistic effects of combining medicinal mushrooms with conventional ophthalmic treatments could lead to more effective and holistic approaches to eye care.
