# Supplementary material for: The therapeutic potential of medicinal mushrooms in ophthalmology: from ethnomycology to evidence-based applications
Source: Front Pharmacol. 2026 Sep 14;17:1887133. doi: 10.3389/fphar.2026.1887133 (PMC13617288; doi:10.3389/fphar.2026.1887133)
Supplement: Supplementary file 1 [file Table1.docx]

***Supplementary Table S1.*** *Self-edited summarizing table about the medicinal mushrooms mentioned in the publication, detailing which mushrooms are used for which eye diseases in evidence-based ophthalmology and Traditional Chinese ophthalmology^a^*

| Scientific name, botanical family | Significant active agents and marker molecules | Pharmacologic properties | Target ophthalmic disease  (and level of evidence)^b^ | References |
| --- | --- | --- | --- | --- |
| *Acremonium chrysogenum* (Thirum. & Sukapure) W. Gams (Ascomycota) |   Cephalosporine core^c^ | Antibiotic effect via inhibition of bacterial cell wall synthesis | Bacterial conjunctivitis (1)  Endophthalmitis prevention and therapy (1) | (Wykoff et al., 2011; Meyer et al., 2021) |
| *Agaricus bisporus* (J. E. Lange) Inbach (Basidiomycota) |   ***A. bisporus* lectin** (ABL) | Dose-dependent anti-fibrotic agent during trabeculectomy (0.1 mg/mL) | Glaucoma (5)  Proliferative vitreoretinopathy (5) | (Batterbury et al., 2002; Kent et al., 2003a, 2003b; Muszyńska et al., 2017) |
|  |  | Inhibits collagen matrix contraction in a concentration-dependent manner |  |  |
|  |  | Anti-fibrotic |  |  |
| *Auricularia heimuer* F. Wu, B.K. Cui, Y.C. Dai (Basidiomycota) |   *Auricularia*-soluble polysaccharides, **scleroglucan**, schizophyllan, lentinan | Thermal stability and gel-forming properties in water (hydrocolloid) | Vitreous hemorrhage (5) | (Kalitukha et al., 2023) |
|  |  | Anti-inflammatory |  |  |
|  |  | Antioxidant |  |  |
|  |  | Anticoagulant |  |  |
| *Cortinarius caperatus* (Pers.) Fr. (Basidiomycota) |   Protein RC28, **phytic acid**, 5-(1-hydroxyethyl) dihydrofuran-2(3H)-one, 4-ethoxy-4-oxobutanoic acid, 4-ketononanoic acid, S-acetyl-N-glycyl cysteamine, methyl benzoate, p-hydroxybenzoic acid | Antiviral effects against herpes simplex virus-1 (HSV-1) | Herpes keratitis (5) | (Yan et al., 2015; Ridwan et al., 2018) |
| *Fusidium coccineum* Corda (Ascomycota) |   **Fusidic acid** | Antibiotic: basteriostatic effect (mRNA synthesis inhibition) | Bacterial conjunctivitis (1) | (Jackson et al., 2002) |
| *Ganoderma lucidum* (Curtis) P. Karst. (Basidiomycota) |   **Ganoderic acid A**, triterpenoids, *G. lucidum* polysaccharides (GLP) | Significantly improves the survival rate and liver function in α-Amatoxin intoxication by inhibition of JAK2-STAT3 pathway | Dry eye disease (5)  Glaucoma (5)  AMD (2) | (Amen et al., 2017; Parravano et al., 2019; Seweryn et al., 2021; Okenwa-Vincent et al., 2023; Rák and Csutak, 2024a; Xiao et al., 2024; Areesanan et al., 2025) |
|  |  | Reducing ROS levels and the secretion of inflammatory cytokines in UVB-exposed human corneal epithelial cells and Meibomian gland cells |  |  |
|  |  | Reduce levels of inflammatory cytokines such as IL-1β and TNF-α, as well as the activity of the NF-κB signaling pathway |  |  |
|  |  | Increased corneal cell proliferation by 25% and accelerated wound healing |  |  |
|  |  | Rho-kinase inhibitory effect (ROCK-I & ROCK-II) |  |  |
|  |  | Significant increase in the mfERG response amplitude density in the central macular areas |  |  |
| *Gloeophyllum odoratum* (Wulfen) Imazeki (Basidiomycota) |   *G. odoratum* triterpenes: **gloeophyllin A**-B, gloeophyllin K-L, trametenolic acid B, eburicodiol, ergosterol peroxide, 21-hydroxilanosterol | Reducing ROS levels and the secretion of inflammatory cytokines in UVB-exposed human corneal epithelial cells and Meibomian gland cells | Dry eye disease (5) | (Cateni et al., 2015; Grienke et al., 2019; Liu et al., 2020; Areesanan et al., 2025) |
|  |  | Thrombin inhibitor (72% at a concentration of 120 μg/mL) |  |  |
| *Grifola frondosa* (Dicks.) Gray (Basidiomycota) |   **Ergothioneine** | Reduction of oxidative stress (lipofuscin, 8-oxoguanine, mitochondrial DNA damage) | AMD (5) | (Ostrovski and Zinner, 2024) |
| *Hericium erinaceus* (Bull.) Persoon (Basidiomycota) |   **Erinacine A** | Anti-inflammatory, neuroprotective | Traumatic optic neuropathy (5) | (Hsu et al., 2023) |
|  |  | Ameliorates flash VEP function, the density of retinal ganglion cells |  |  |
|  |  | Reduced the levels of oxidative stress markers detectable by Western blot (pRIP1, Cas8, cCas3, TNF-α, TNFR1, IL-1β, iNOS), while increasing the levels of antioxidant enzymes (Nrf2, HO-1, SOD1) |  |  |
| *Isaria cicadae* Miq. (Ascomycota) |   **N6-(2-hydroxyethyl) adenosine**, cordicepin, cyclic heptapeptide, mannitol, ergosterol | Immunomodulatory | Dry eye disease (2)  Glaucoma (5)  Cataract (5) | (Lin et al., 2017; Hong et al., 2021; Chang et al., 2022; Lu et al., 2023) |
|  |  | Reduced levels of inflammatory markers such as IL-1β and TNF-α in the tear film |  |  |
|  |  | Improved tear film osmolarity and increased TBUT values |  |  |
|  |  | No significant change in tear production |  |  |
|  |  | Reduces apoptosis of corneal epithelial cells and maintains conjunctival goblet cells |  |  |
|  |  | Significant reduction in malondialdehyde levels and an increase in the activity of antioxidant enzymes such as superoxide dismutase and catalase |  |  |
|  |  | Decreased the expression of heat-shock proteins associated with UVB induced cataract formation |  |  |
| *Isaria sinclairii* (Berk.) Lloyd (Ascomycota) |   **Fingolimod**, myriocin (thermozymocidin), mycestericin A-G | Oral dose of 0.1 mg/kg or higher significantly prolongs allograft survival in various experimental allotransplantation models and autoimmune disease model | Relapsing multiple sclerosis (1) | (Chiba, 2020; Wang et al., 2022) |
|  |  | Phospholipoid mediator (“functional” antagonist at S1P_1_ receptor) |  |  |
| *Lentinula edodes* (Berk.) Pegler (Basidiomycota) |   **Lentinan**, β-glucans, syringic acid and vanillic acid | Immunomodulator and anti-inflammatory: reduce the production of proinflammatory cytokines (IL-2, IL-6, IL-8, and TNF-α), while markedly enhancing the secretion of the anti-inflammatory cytokine IL-10 | Chronic anterior uveitis (5) | (Dai et al., 2023) |
|  |  | Antioxidant |  |  |
| *Monascus purpureus* Went (Ascomycota) |   **Lovastatin** | Dose-dependent anti-fibrotic agent during trabeculectomy (0.1 mg/mL) | Glaucoma (5) | (Park et al., 2016) |
| *Penicillium brevicompactum* Dierckx (Ascomycota) |   **Mycophenolate mofetil** | Prodrug of mycophenolic acid depleting guanosine triphosphate and suppresses the de novo synthesis of purines, thus inhibiting proliferation and inducing apoptosis of activated T-lymphocytes | Endocrine orbitopathy (1)  Ocular pemphigoid (1) Autoimmune uveitis (1)  Scleritis (1)  Ocular surface inflammations (1) | (Daniel et al., 2010; Bartalena et al., 2021; Feng et al., 2022) |
| *Penicillium griseofulvum*  Dierckx (Ascomycota) |   Patulin, penifulvin A, cyclopiazonic acid, roquefortine C, shikimic acid, **griseofulvin**, and 6-methylsalicylic acid | Off-target inhibition of ferrochelatase: possible antiangiogenic effect | AMD (wet) (5)  Diabetic retinopathy (5) | (Corson et al., 2016, 2023; Chobisa et al., 2023) |
| *Penicillium rubens* Biourge (Ascomycota) |   **Penicillin-G,** penicillactone A-B | Potent bactericide effect especially towards *Treponema pallidum* | Ocular syphilis (1) | (Houbraken et al., 2011; Géhl et al., 2020; Liu et al., 2020; Gyéresi and Papp, 2022) |
| *Pleurotus ostreatus* (Jacq.) Quél. (Basidiomycota) |   *P. ostreatus* polysaccharides (POP), lovastatin, pleuran, **asperpyrone** A-**F**, fonsecinone A-B, aurasperones A-E, cumene, butylated hydroxytoluene, acetophenone, glutamic acid | Dose-dependent IOP reduction similarly to timolol | Glaucoma (5)  Cataract (5) | (Isai et al., 2009; Ganeshpurkar et al., 2011; Ebigwai et al., 2012; Cai et al., 2019; Zhao et al., 2024b) |
| *Pleurotus ostreatus* var. *florida* Cetto (Basidiomycota) |  | Delay of selenite-induced or hyperglycaemia induced cataractogenesis by antioxidant effect |  |  |
|  |  | Reduced malondialdehyde levels and increased catalase and glutathione levels |  |  |
|  |  | Immunomodulatory effect |  |  |
| *Pleurotus tuber-regium* (Rumph. ex Fr.) Singer (Basidiomycota) |   β-glucans, **D-mannitol**, phytic acid, glucuronic acid | Dose-dependent IOP reduction similarly to latanoprost, D-mannitol and acetazolamide (inhibiting the activity of angiotensin1  converting enzyme) | Glaucoma (5) | (Akinlabi and Erhabor, 2016; Lin et al., 2020; Odjimogho et al., 2024) |
| *Polyporus umbellatus* (Pers.) Fr. (Basidiomycota) |   ***P. umbellatus* polysaccharide** (PUP/PPS), *P. umbellatus* acidic polysaccharide (GU-1), PUP60S2, PUP60W-1, PUP80S1, GUMP-1-1 and GUMP-1-2 | Effects on aquaporin channels and the downregulation of TGF-β1 and Smad | Glaucoma (5)  AMD (wet) (5)  Berlin’s oedema (5)  Hypertensive retinopathy (5) | (Yang et al., 2018; Jiang et al., 2020; Ou et al., 2020; Liu et al., 2021; Li et al., 2022) |
|  |  | Diuretic |  |  |
|  |  | Antifibrotic |  |  |
| *Terfezia claveryi* Chatin (Ascomycota) |   Terfezia lectin, linoleic acid, **stigmasterol**, β-sitosterol, squalene, lupeol, p-cymene, carveol, limonene, guaiene, and cumene hydroperoxide | Bacteriostatic to *Chlamydia trachomatis* | Trachoma (5) | (Gouzi et al., 2011; El Enshasy et al., 2013; Dahham et al., 2018) |
| *Terfezia boudieri* Chatin (Ascomycota) |  | Significantly inhibited the growth of *Staphylococcus aureus* and *Pseudomonas aeruginosa* |  |  |
| *Tirmania nivea* (Desf.) Trappe (Ascomycota) |  | Antiangiogenic |  |  |
|  |  | Antioxidant |  |  |
|  |  | Antiapoptotic |  |  |
| *Theissenia cinerea* Y.M. Ju, J.D. Rogers & H.M. Hsieh (Xylariaceae) |   **Theissenolactone C** (LC53) | Inhibiting NF-κB and MMP-9 activity following high-IOP-induced retinal damage | Glaucoma (5) | (Lin et al., 2019; Rák and Csutak, 2024a) |
| *Tolypocladium inflatum* W. Gams (Ascomycota) |   **Cyclosporin-A** | Inhibits apoptosis of ocular surface cells induced by inflammatory biochemical pathways and migration of T-lymphocytes to the ocular surface | Severe immune-mediated ocular surface inflammation and dry eye associated with Sjögren’s syndrome, systemic lupus erythematosus, systemic sclerosis, rheumatoid arthritis, and vernal keratoconjunctivitis (1) | (Yagci and Gurdal, 2014; Chiba, 2020; Aragona et al., 2021; Rák and Csutak, 2024b) |
| *Tremella fuciformis* Berk. (Basidiomycota) |   **Tremella fuciformis polysaccharide** | Similar structure to hyaluronic acid, non-toxic, easy solubility in water, and good colloidal properties and stability | Vitreous hemorrhage (5) | (Colomer et al., 2016; Chandra et al., 2022; Wan et al., 2025) |
|  |  | Moisturizing effect |  |  |
|  |  | Reduce cerebral edema and decrease neuronal apoptosis by inhibiting the KDR-mediated P38 MAPK/NF-κB pathway |  |  |
| *Wolfiporia extensa* (Peck) Ginns (Basidiomycota) |   **Poricoic acid A**, Poria cocos polysaccharides (PCP), carboxymethyl polysaccharide (CMP33), polyporic acid C, trametenolic acid, tumulosic acid, pachymic acid, and dehydroeburictrametoic acid | Effects on aquaporin channels and the downregulation of TGF-β1; Smad; NF-κB /MAPK; Wnt/β-catenin; and AMPK pathways | Glaucoma (5)  AMD (dry and wet) (5)  Cataract (5)  Berlin’s oedema (5)  Hypertensive retinopathy (5)  Endocrine orbitopathy (5)  CSCR (5)  Chalazion (5)  Muscae volantes (5)  Vitreous hemorrhage (5)  Chronic anterior uveitis (5) | (Yang et al., 2018; Ou et al., 2020; Li et al., 2022; Guo et al., 2024; Zhao et al., 2024a) |
|  |  | Increase ERK/Nrf2/HO-1 signaling pathway |  |  |
|  |  | Diuretic, decreasing edema |  |  |
|  |  | Decreased ROS and malondialdehyde levels, and increase SOD activity |  |  |
|  |  | Reduces NO release and cytokine secretion (IL-1β, IL-6 and TNF-α) |  |  |
|  |  | Reduces PGE levels by downregulating COX-2 |  |  |
|  |  | VEGF-inhibitor |  |  |

^a^Sources are detailed in text and references.

^b^Level of Evidence-based on the Oxford Centre for Evidence-Based Medicine (OCEBM) guidelines: Level 1: Systematic reviews and meta-analyses of randomized controlled trials (RCTs); Level 2: Individual RCTs and robust cohort studies; Level 3: Case-control studies; Level 4: Case series and poor-quality cohort/case-control studies; Level 5: Mechanism-based reasoning, expert opinion, and preclinical (in vitro/in vivo) studies.

^c^Chemical structures of the highlighted molecules are presented; images were obtained from MedChem.

Abbreviations: AMPK – AMP‑activated protein kinase; AMD – Age‑Related Macular Degeneration; Cas8 – CRISPR‑associated protein 8; cCas3 – catalytically active CRISPR‑associated protein 3; CNV – choroidal neovascularization; COX‑2 – cyclooxygenase‑2; DNA – deoxyribonucleic acid; ERK – extracellular signal‑regulated kinase; GLP – Ganoderma lucidum polysaccharides; HO‑1 – heme oxygenase‑1; HSV‑1 – Herpes simplex virus‑1; IL – interleukin; iNOS – inducible nitric oxide synthase; IOP – intraocular pressure; JAK2 – Janus kinase 2; MAPK – mitogen‑activated protein kinase; mfERG – multifocal electroretinogram; MMP‑9 – matrix metalloproteinase 9; NF‑κB – nuclear factor kappa B; Nrf2 – nuclear factor erythroid 2‑related factor 2; pRIP1 – phosphorylated receptor‑interacting protein 1; PGE – prostaglandin E; ROS – reactive oxygen species; S1P1 – sphingosine‑1‑phosphate receptor 1; SOD1 – superoxide dismutase 1; TGF‑β1 – transforming growth factor beta 1; TNF‑α – tumor necrosis factor alpha; TNFR1 – tumor necrosis factor receptor 1; VEGF – vascular endothelial growth factor; VEP – visual evoked potentials; Wnt – Wingless/Integrated signaling pathway.
